# Novel High Isolation and High Capacitance Ratio RF MEMS Switch: Design, Analysis and Performance Verification

**DOI:** 10.3390/mi13050646

**Published:** 2022-04-19

**Authors:** Zhongliang Deng, Yucheng Wang, Kun Deng, Chengqi Lai, Jiali Zhou

**Affiliations:** 1 School of Electronic Engineering, Beijing University of Posts and Telecommunications, Haidian District, Beijing 100876, China; dengzhl@bupt.edu.cn (Z.D.); laichengqi@bupt.edu.cn (C.L.); 2 School of Automation, Beijing University of Posts and Telecommunications, Haidian District, Beijing 100876, China; dengkun@bupt.edu.cn (K.D.); zhoujiali@bupt.edu.cn (J.Z.)

**Keywords:** RF MEMS, high isolation, low insertion loss, high capacitance ratio

## Abstract

In this paper, a novel high isolation and high-capacitance-ratio radio-frequency micro-electromechanical systems (RF MEMS) switch working at Ka-band is designed, fabricated, measured and analyzed. The proposed RF MEMS switch mainly consists of a MEMS metallic beam, coplanar waveguide (CPW) transmission line, dielectric layer and metal–insulator–metal (MIM) fixed capacitors. The measured results indicate that the insertion loss is better than 0.5 dB at 32 GHz, and the isolation is more than 35 dB at the resonant frequency. From the fitted results, the capacitance ratio is 246.3. Compared with traditional MEMS capacitive switches, this proposed MEMS switch exhibits a high capacitance ratio and provides a wonderful solution for cutting-edge performance in 5G and other high-performance applications.

## 1. Introduction

Compared with PIN diodes and FETs, RF MEMS switches are smaller in size, lighter in weight, less sensitive to acceleration, no DC power at microwave frequencies and have excellent isolation and insertion loss [1]. Grant et al. [2] address the fundamentals of RF switches, providing a comparison between semiconductor and RF MEMS switches. RF MEMS switches are widely used in radar [3], satellite communication systems [4], smart antennas with beam forming and phased array capabilities [5], tunable filters [6], phase-shifting networks [7] and other important fields. Daneshmand et al. [4] describe the potential applications of RF MEMS switch matrices in the satellite industry, wherein mass reduction and performance improvement are crucial. J. Rodriguez [5] tells us that RF MEMS switches and transmission lines can realize the function of phase shift so that they can point out the antenna beam to a desired location with high precision.

However, there are a few issues to be resolved. First, the driving voltage of the MEMS switch increases as the beam height increases. Secondly, the capacitance ratio is not very high. The actuation voltage represents the switch’s integration performance, which contributes to the development of a monolithic microwave-integrated circuit (MMIC). A larger off/on capacitance ratio is advantageous for achieving high isolation and outstanding RF performance. For example, the capacitance ratio of the switch controls the adjustable range of the center frequency of the resonant unit in the tunable filter.

There has been some research done in larger capacitance off/on ratios to date. Al-Dahleh R et al. [8] employ warped-beam, and Angira et al. [9] and Persano et al. [10] use materials with a high dielectric constant to get a larger capacitance ratio. The other approach of achieving a high capacitance ratio is to widen the distance between the MEMS beam and the dielectric layer [11]. However, the capacitance ratio is constrained by the minimum dielectric layer thickness, the maximum dielectric constant value and the maximum distance between the beam and the signal transmission line. As a result, the ways used in [8,9,10,11,12] are not the most suitable.

Based on the floating metal membrane, this research increases the capacitance ratio of the RF MEMS switch. Therefore, a low insertion loss, high isolation and larger capacitance ratio RF MEMS switch has been designed and fabricated.

## 2. Materials and Methods

### 2.1. Circuit Topology and Theory Analysis

**Figure 1:**
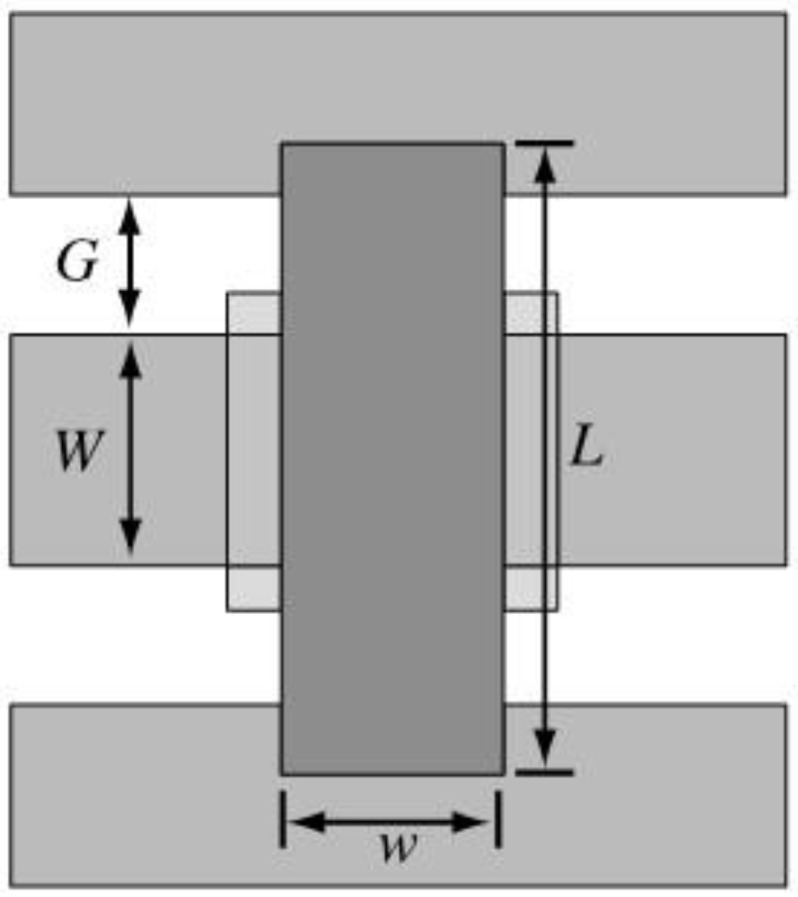
MEMS capacitive parallel switch top view.

MEMS parallel switches can be integrated in coplanar waveguides (CPW) or microstrip lines. In this paper, 50 Ω CPW is used to making the circuit model analysis. CPW is generally defined by center strip width W and gap width G. The CPW transmission line has a dielectric layer of thickness *t_d_*, and the dielectric constant is *ε_r_*. The length of the beam is *L*; the width is *w*, and the thickness is *t*. A MEMS capacitive parallel switch top view is shown in Figure 1.

**Figure 2:**
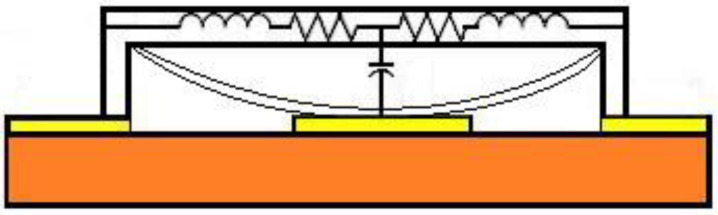
Model of a MEMS switch in a working state.

**Figure 3:**
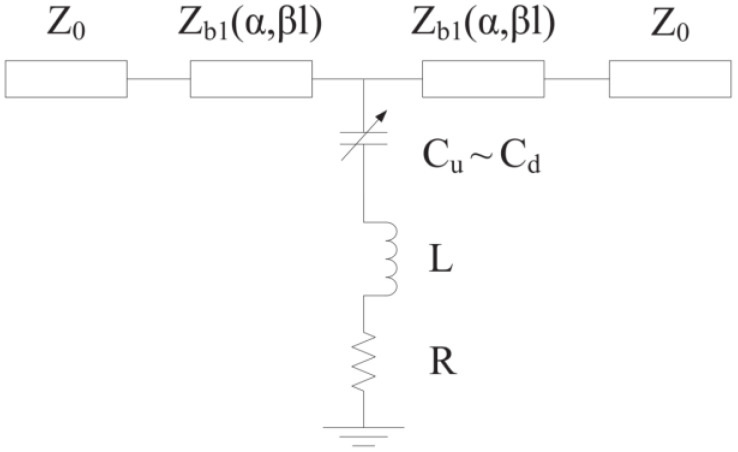
Circuit topology of traditional MEMS switches.

A common MEMS parallel switch beam is connected between the transmission line and ground, and the switch anchor is connected to the CPW ground. Figure 2 shows the equivalent model of the switch in operation, wherein the beam mainly produces inductance, and the beam with the transmission line and the middle of the dielectric produces the capacitor. When the DC voltage is applied between the MEMS beam and the CPW transmission line, the resulting electrostatic force causes the beam to bend downward and switches to the down-state. When the DC bias voltage is not applied, the beam returns to the initial state (up-state) by the elastic restoring force. Figure 3 shows the circuit topology of the typical MEMS switch.

The wave port of the characteristic impedance is *Z*_0_. *Z_b_*_1_ (*α*, *βl*) denotes the characteristic impedance of the transmission line between the wave port and the edge of the MEMS beam; *α* is the transmission attenuation constant, and *βl* denotes electric length of the transmission line. The capacitive ratio *C_r_* and the actuation voltage *V_p_* of the MEMS switch can be calculated from Formula (1) and (2).

(1) $$C_{r}=\frac{C_{d}}{C_{u}}=\frac{\frac{\varepsilon_{0}\varepsilon_{r}A}{t_{d}}}{\frac{\varepsilon_{0}A}{g+\frac{t_{d}}{\varepsilon_{r}}}+C_{f}}$$

(2) $$V_{p}=\sqrt{\frac{8kg^{3}}{27\varepsilon_{0}A}}$$

where parameters of *C_d_*, *C_u_*, *ε_0_*, *ε_r_*, *t_d_*, *g*, *C_f_*, *A* and *k* are the down-state capacitance of the MEMS switch, the up-state capacitance of the MEMS switch, vacuum dielectric constant, relative dielectric constant of the dielectric layer, the thickness of the dielectric layer, the gap between the beam and the electrodes, the edge capacitance of the up-state capacitance, the cross-sectional area of the electrode and the spring constant of the beam, respectively.

**Figure 4:**
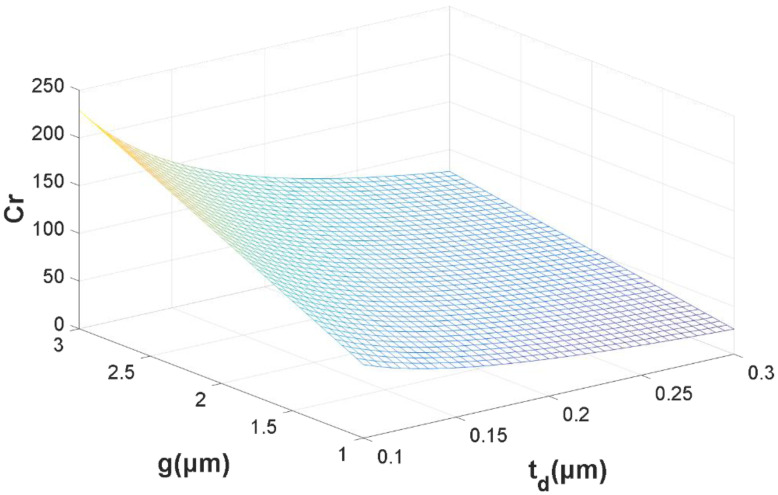
The relationship between *g*, *t_d_* and *C_r_*.

When *C_f_* is not taken into account, the Formula (1) becomes Equation (3). From equation (3), we can use MATLAB R2015a (MathWorks, Natick, MA, USA) to obtain Figure 4, which is about the relationship between *g*, *t_d_* and *C_r_*.

(3) $$C_{r}=\frac{C_{d}}{C_{u}}=\frac{\frac{\varepsilon_{0}\varepsilon_{r}A}{t_{d}}}{\frac{\varepsilon_{0}A}{g+\frac{t_{d}}{\varepsilon_{r}}}}=\frac{g\varepsilon_{r}}{t_{d}}+1$$

As a result, the pull-in voltage *V_p_* can be simplified from (3)–(5).

(4) $$g=\frac{\left(C_{r}-1\right)t_{d}}{\varepsilon_{r}}$$

(5) $$V_{p}=\sqrt{\frac{8k{\left(C_{r}-1\right)}^{3}t_{d}{}^{3}}{27\varepsilon_{0}\varepsilon_{r}{}^{3}A}}$$

From Equation (3) and Figure 4, we may deduce that the capacitive ratio *C_r_* rises as *g* (the gap between the beam and the electrodes) rises and falls as *t_d_* (the thickness of the dielectric layer) rises. However, due to the pinhole problems in the dielectric layer, we can not deposit a Si_3_N_4_ layer less than 0.1 μm, and the dielectric layer should be able to sustain the excitation voltage without being pierced. Therefore, it is difficult to achieve low pull-in voltage and a larger capacitance ratio for traditional MEMS switches simultaneously.

**Figure 5:**
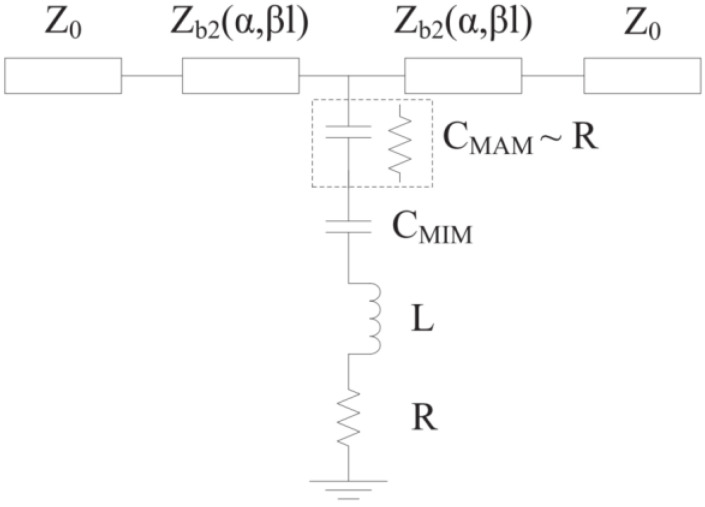
Circuit topology of the proposed MEMS switch with MIM capacitors.

As illustrated in Figure 4, when *g* = 1.5 μm, Cr is less than 80 for typical MEMS switches using the circuit model of Figure 3. Connecting a MIM capacitor to the shunt capacitor C_metal–air–metal (MAM)_ is a simple and practical way to get a high capacitance ratio in a MEMS switch, as shown in Figure 5. A MIM capacitor, which acts as a shunt capacitor on one side of the CPW ground plane, was proposed in [12]. In this article, a MIM capacitor is situated below the beam. The upper metal plate of the MIM capacitor, the beam and the air combine to form a MAM capacitor. When the MEMS switch is in the up-state, a MIM capacitor is connected in series to a MAM capacitor. On the contrary, the MAM capacitor is transformed to a resistance R when the MEMS switch is in the down-state.

Define *λ* = *A_MIM_/A_MAM_*. *C_r_* can be calculated as follows, when the edge capacitances of the up-state capacitance and the down-state capacitance are neglected.

(6) $$C_{\mathrm{MAM}}=\frac{\varepsilon_{0}}{g}A_{MAM}$$

(7) $$C_{\mathrm{MIM}}=\frac{\varepsilon_{0}\varepsilon_{r}}{t_{d}}A_{MIM}$$

(8) $$C_{r}=\frac{C_{d}}{C_{u}}=\frac{C_{\mathrm{MIM}}}{C_{\mathrm{MIM}}||C_{\mathrm{MAM}}}=\frac{C_{\mathrm{MIM}}}{C_{\mathrm{MAM}}}+1=\frac{\varepsilon_{r}g}{t_{d}}\frac{A_{MIM}}{A_{MAM}}+1=\frac{\varepsilon_{r}g}{t_{d}}\lambda +1$$

**Figure 6:**
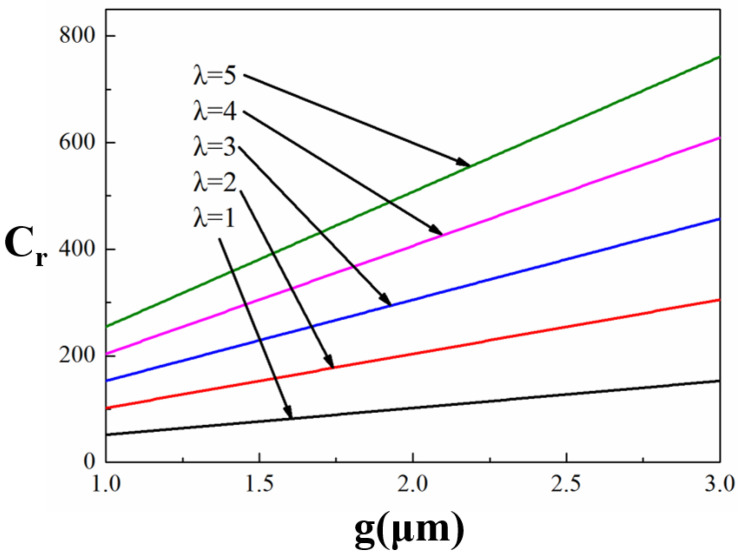
The relationship between the parameters of *g*, *Cr* and *λ*.

Figure 6 shows the relationship between the parameters of *g*, *C_r_* and *λ*, when *k* = 20 N/m, *t_d_* = 0.15 μm and *ε_r_* = 7.6. It has been discovered that *C_r_* increases with the increase of *λ* and *g*.

### 2.2. Spring Constant and Actuation Voltage Analysis

**Figure 7:**
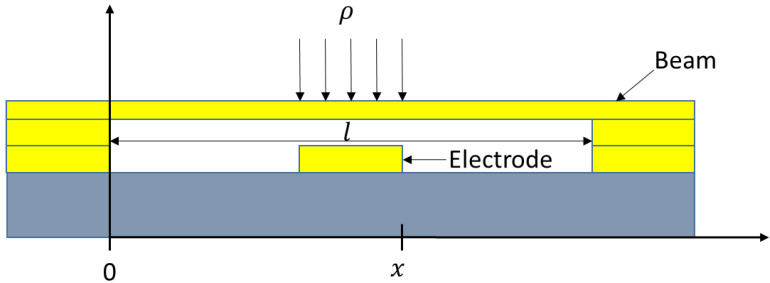
Electrode topology of the MEMS switch.

As shown in Figure 7, the electrode sits on the signal of the CPW. MEMS switches have a spring constant *k*, which may be calculated as follows.

(9) $$k_{1}=-\frac{P}{\frac{2}{EI}\int_{\frac{l}{2}}^{x}\frac{\rho }{48}\left(l^{3}-6l^{2}a+9la^{2}-4a^{3}\right)da}$$

(10) $$k_{2}=-\frac{P}{2\int_{\frac{l}{2}}^{x}\frac{\rho }{2S}\left(l-a\right)da}$$

(11) $$k=k_{1}+k_{2}$$

(12) $$I=\frac{wt^{3}}{12}$$

(13) $$P=2\rho \left(x-\frac{l}{2}\right)$$

(14) S= σ(1−v)tw

where the spring constant $k_{1}$ is the spring constant caused by the stiffness of the beam, $k_{2}$ is the spring constant caused by biaxial residual stress of the beam, ρ is the uniformly distributed load of the beam, S is the tensile force of biaxial residual stress, σ is biaxial residual stress, v is the Possion ratio, E is the Young’s modulus, *l* is the length of the beam, *w* is the width of the beam, and *t* is the thickness of the beam.

The actuation voltage of the MEMS switch can be calculated as follows:

(15) $$V_{p}=\sqrt{\frac{8k}{27\varepsilon_{0}\left(2\left(x-\frac{l}{2}\right)\right)w}g_{0}^{3}}$$

**Figure 8:**
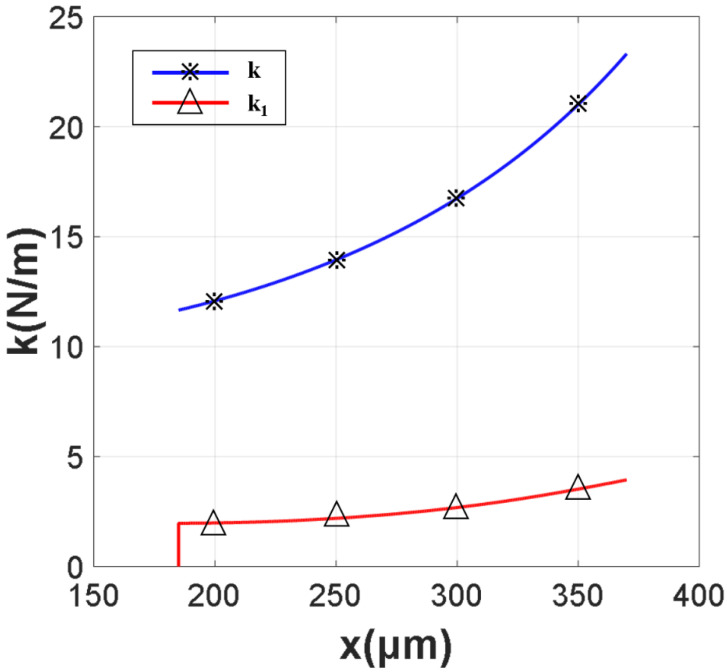
The relationship between x and $k_{1}$, k.

Figure 8 depicts the relationship between the spring constant and the electrode coordinates. From Figure 8, we can observe that, as the area of the electrode decreases, the spring constant decreases. For the rectangle beam with Au (*E* = 78 Gpa, *v* = 0.44), the length of the beam is 370 μm, the width of the beam is 160 μm, the thickness of the beam is 1 μm, $g_{0}$ (the gap between beam and CPW) is 2 μm and σ=10 Mpa.

**Figure 9:**
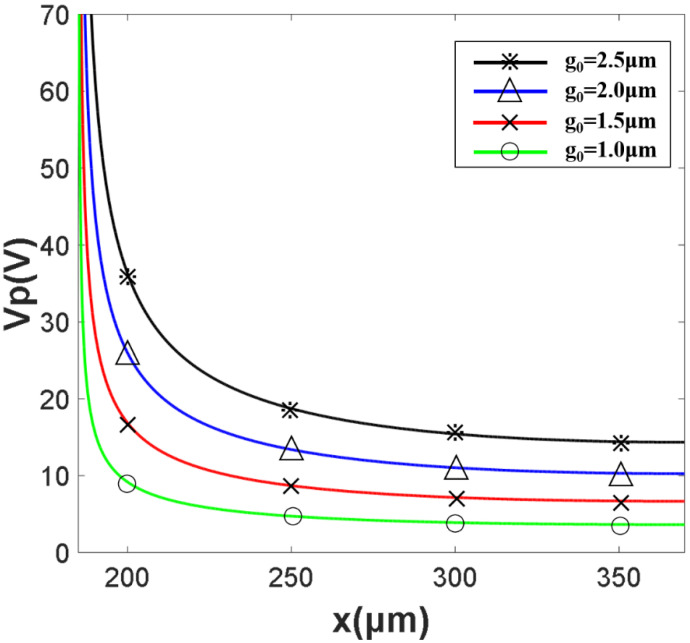
The relationship between the x, $g_{0}$ and $V_{p}$.

Figure 9 shows the actuation voltage $V_{p}$ is changed with the change of x and $g_{0}$. When $g_{0}$ = 2 μm, x ≥ 235 μm, $V_{p}$ ≤ 15 V. When $g_{0}$ = 1.5 μm, x ≥ 235 μm, $V_{p}$ ≥ 10 V. When $g_{0}$ = 1 μm, x ≥ 235 μm, $V_{p}$ ≤ 6.5 V.

### 2.3. Design of the High Capacitance Ratio RF MEMS Switch and Simulation

**Figure 10:**
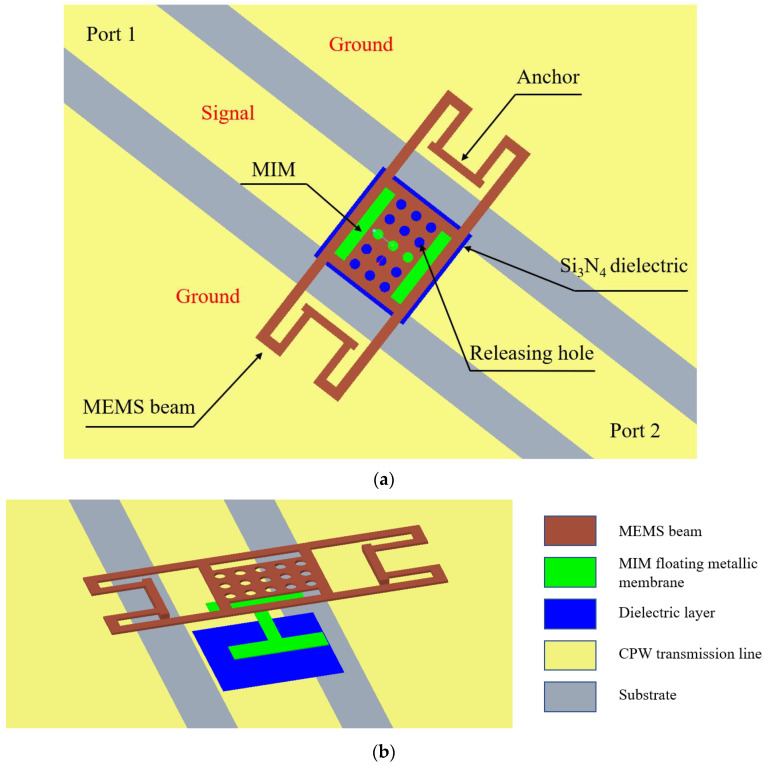
(**a**) The top view of the proposed MEMS switch; (**b**) The dismantled figure of the proposed MEMS switch.

The proposed high capacitance ratio MEMS switch is shown in Figure 10a,b. It includes a serpentine flexure MEMS metallic beam, metal–insulator–metal (MIM) floating metallic membrane, dielectric layer and a CPW transmission line.

Table 1 shows the material of each part of the MEMS switch.

**Figure 11:**
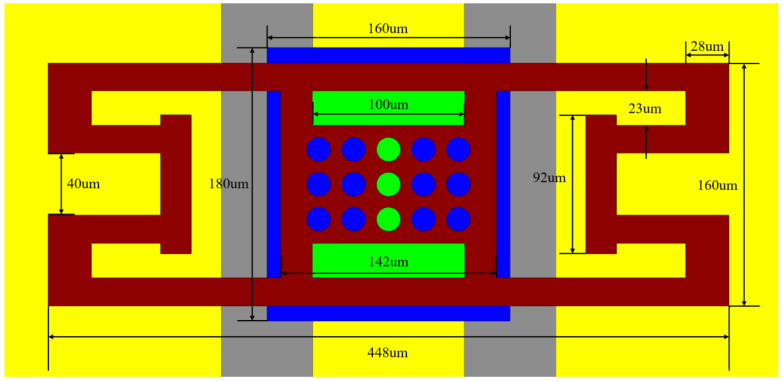
Physical dimensions of the suggested RF MEMS switch.

The physical dimensions of the suggested RF MEMS switch are shown in Figure 11. Four serpentine springs are coupled to the RF MEMS switch beam.

**Figure 12:**
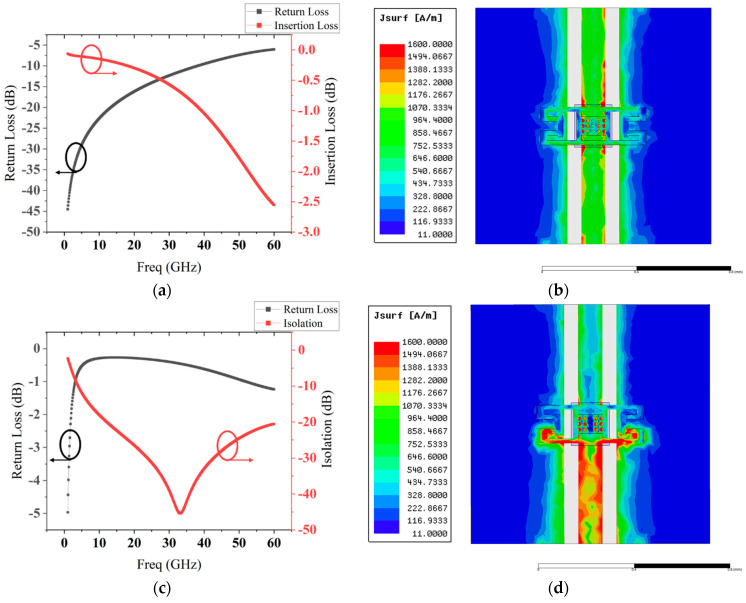
Simulated S parameters and current distribution diagrams: (**a**,**b**) up-state; (**c**,**d**) down-state.

Then we use the HFSS software to model and simulate the switch through the above structure. Figure 12 shows the simulated S parameters and current distribution diagrams. From Figure 12a,c, we can draw a conclusion that the insertion loss is better than 0.5 dB at 30 GHz when the switch is in the up state and the isolation is more than 42 dB at the resonant frequency in the down-state. Current distribution diagrams shown in Figure 12b,d demonstrate the remarkable signal isolation capacity of the MEMS switch.

## 3. Fabrication, Measurements and Discussions

### 3.1. Fabrication

The specific main process flow can be divided into nine steps:

1. The whole structure of the proposed high-isolation RF MEMS switch was fabricated on a high-resistivity silicon substrate with a thickness of 400 µm. At the bottom of the substrate is 0.2-μm-thick Au.
2. SiO_2_ (0.3-μm-thick), which acts as an insulating layer, is grown on the substrate by means of thermal oxidation. The SiO_2_ layer can increase the adhesion of materials used in subsequent processes.
3. The high-resistance DC bias lines are sputtered and patterned, and 0.16-μm-thick Si_3_N_4_ is deposited on top of the bias lines.
4. The CPW transmission line consists of a 0.2-μm-thick Au center conductor and 2-μm-thick Au ground planes.
5. The bottom electrode is covered with a 0.16-μm-thick Si_3_N_4_ layer, which is deposited using the plasma-enhanced chemical vapor deposition (PECVD) for DC isolation.
6. A total of 0.2 µm Au was evaporated as the MIM floating metallic membrane.
7. After the thermal curing process, a 2-μm-thick polyimide is used as the sacrificial layer.
8. The anchor and beam are formed by electroplating for 1 h.
9. A supercritical release method is used to release the polyimide sacrificial layer, so that the MEMS switch beam is be suspended.

**Figure 13:**
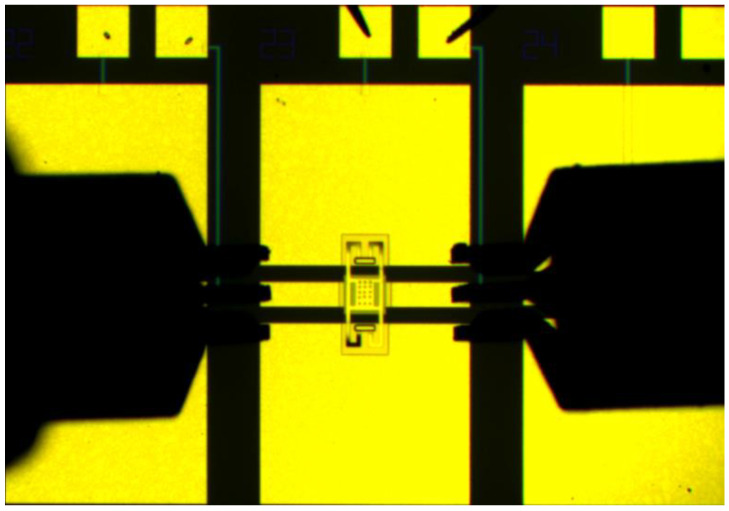
The top view photograph of the proposed RF MEMS switch.

Figure 13 shows the high-isolation RF MEMS switch obtained after processing.

### 3.2. Measurement and Results

#### 3.2.1. Insertion Loss and Isolation

When the RF MEMS switch is in the up-state (namely ON state), the insertion loss displays the signal loss, and when the RF MEMS switch is in the down-state, the isolation shows the signal isolation level (namely OFF state). The S_21_ value between the input and output can be used to determine isolation and insertion loss.

**Figure 14:**
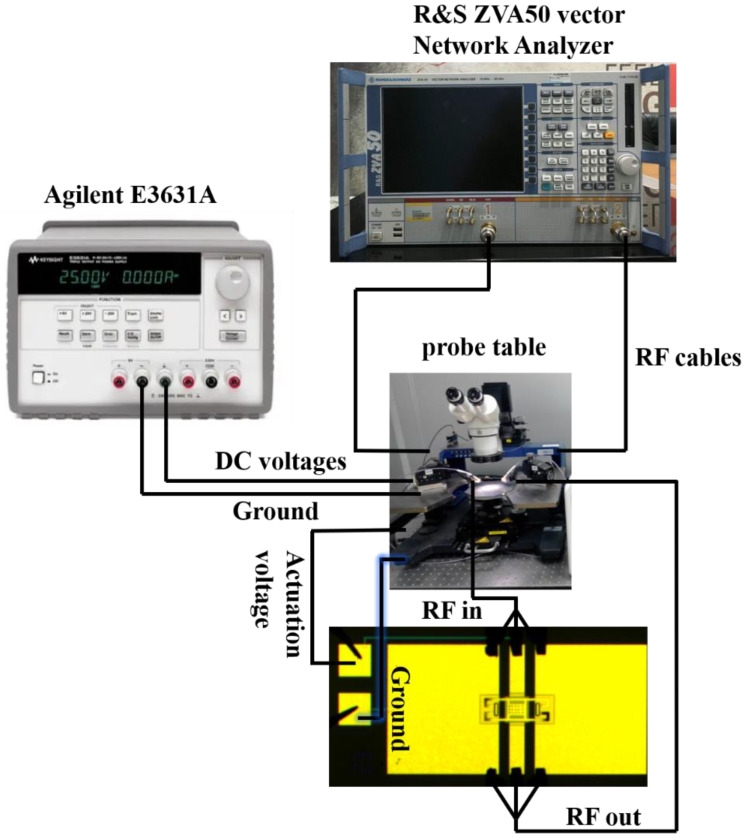
The RF measurement setup of the switch.

The measurements were conducted in an ultraclean room. The temperature and relative humidity of the measurement environment were 24 °C and 40%, respectively. An Agilent E3631A provided the DC voltages. The S (S_11_ and S_21_) characteristics of the RF MEMS switch were measured using a vector Network Analyzer (R&S ZVA50, Rohde & Schwarz, Munich, Germany). To contact the two ends of the switch, two gold ACP-A-GSG-150 probes (Cascade Microtech, Beaverton, OR, USA) were utilized, and the device was put on a probe table (Cascade Summit 11000B-M, Cascade Microtech, Beaverton, OR, USA). The sweep frequency ranged between 10 MHz and 40 GHz. Figure 14 shows the RF measurement setup of the switch.

**Figure 15:**
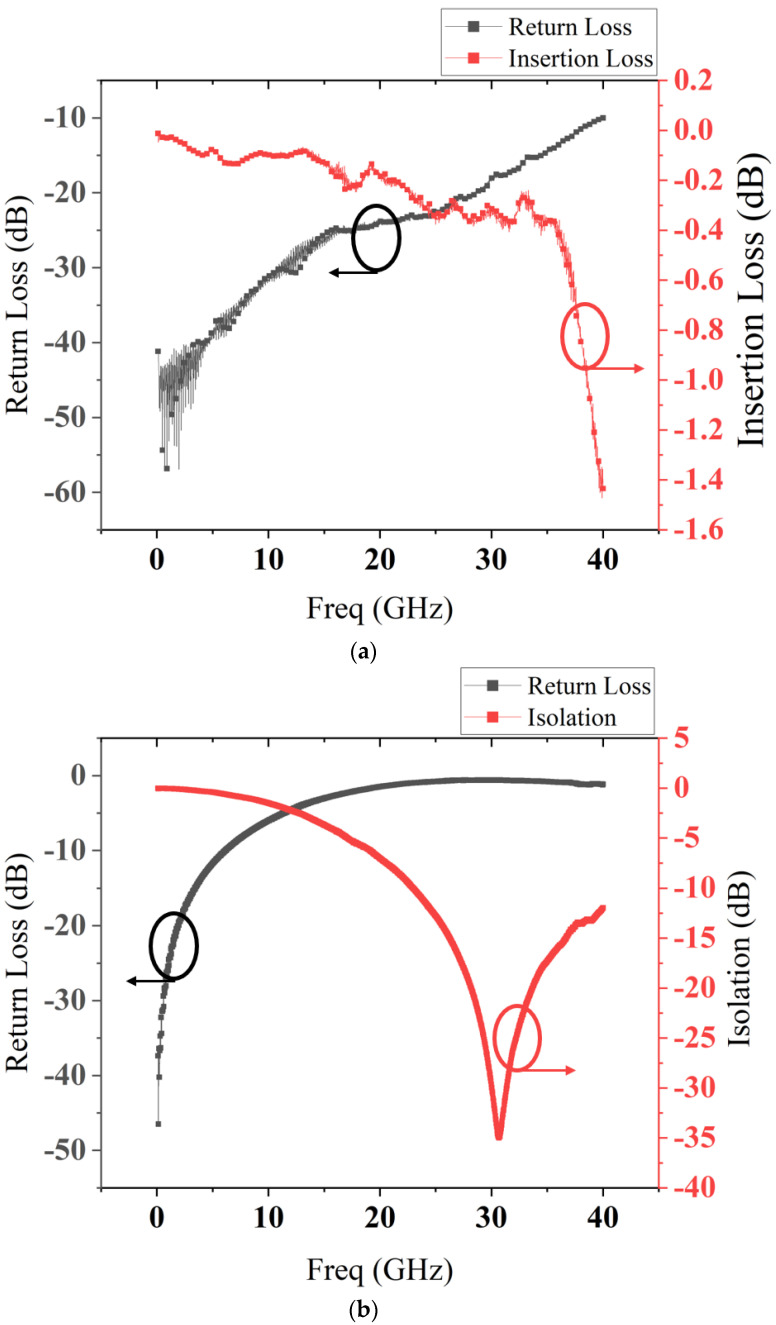
Measurement S parameters results of the proposed RF MEMS switch: (**a**) up-state; (**b**) down-state.

The measured S parameters are shown in Figure 15.

The measured results show that the insertion loss is better than 0.5 dB at 32 GHz, and the isolation is more than 35 dB at the resonant frequency in the range of 25–35 GHz. Compared with simulated results in Figure 12a,c, the measured results match well with simulated results.

#### 3.2.2. Capacitance Ratio

A critical parameter of the proposed RF MEMS switch is the capacitance ratio *C_r_*. However, the up- and down-state capacitance value *C_u_* and *C_d_* are difficult to measure. Measured S parameter extraction was used to obtain the capacitance ratio *C_r_* in this paper. Mathematically, the transmission (*ABCD*) parameters of the proposed circuit model can be expressed as follows:

(16) $$\left(\begin{array}{cc} A & B\\ C & D \end{array}\right)=M_{1}M_{2}M_{1}$$

(17) $$M_{1}=\left(\begin{array}{cc} \cos\theta & jZ_{0}\sin\theta \\ j\frac{1}{Z_{0}}\sin\theta & \cos\theta \end{array}\right)$$

(18) $$M_{2}=\left(\begin{array}{cc} 1 & 0\\ Y_{2} & 1 \end{array}\right)$$

(19) $$Y_{2}={\left[{\left(j\omega C_{s}\right)}^{-1}+j\omega L+R_{s}\right]}^{-1}$$

(20) $$L=\frac{1}{\omega C_{s}}$$

where *M*_1_ denotes the CPW transmission line part, *M*_2_ represents the lumped parameter model of the RF MEMS, θ is the electric length of CPW transmission line, $Z_{0}$ is the characteristic impedance of transmission line and $C_{s}$ is *C_u_* or *C_d_* when the RF MEMS switch stays at different state. S_21_ is:

(21) $$S_{21}=\frac{2}{A+\frac{B}{Z_{0}}+CZ_{0}+D}$$

*C_u_* and *C_d_* can be calculated by using the above equations. *C_u_* = 47.1 fF, *C_d_* = 11.6 pF, *C_r_* = 246.3, $R_{s}\;$= 0.55 Ω. A typical MEMS switch has a capacitance ratio of roughly 100. Therefore, the capacitance ratio in this paper is better than the traditional design.

#### 3.2.3. Actuation Voltage

The actuating voltage of the can be calculated by Formula (15).

The calculated value of effective elastic coefficient *k* is 15 N/m, and the actuating voltage *V_p_* is approximately 13 V. However, the measurement of actuating voltage is 16 V, which is different from the calculation value using Formula (15). The inhomogeneity of thickness and the inadequate release of polyimide are the main causes. The actuating voltage will decrease to the evaluating value when the manufacturing process has a good release and flatness.

#### 3.2.4. Lifespan

**Figure 16:**
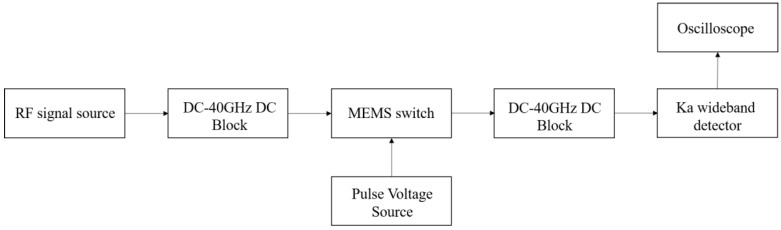
Block diagram of RF MEMS switch lifespan test platform.

The equipment and connection employed to test lifespan are displayed in Figure 16.

The RF MEMS switch is placed on the microwave probe table, and the CPW transmission line port is connected to the RF signal generator and Ka band wideband detector respectively through the GSG microwave probe and the microwave coaxial line. It should be noted that when the CPW transmission line is used as the DC bias electrode of the switch, a DC ~40 GHz block needs to be added to prevent the DC signal from entering the signal generator and detector. The oscilloscope is used to observe and record the pull in and release time of MEMS switch.

The lifespan test of the RF MEMS switch in this paper is carried out by using the above method. We set the rectangular square wave DC voltage frequency to 1 KHz. After 13 h (the RF MEMS switch operates 4.68 × 10^7^ times), the resonant frequency of the switch is always in Ka band. When the switch works for 35 h (1.26 × 10^8^ times), the isolation of MEMS switch in Ka band is less than 10 dB, so that the Ka band detector has difficulty detecting the signal power level when the switching state is changed.

From the test results of switch lifespan, it is found that the resonant frequency of the switch changes as the switching times increase. However, when the resonant frequency of the switch increases to the frequency above Ka band, the switch has no mechanical failure and dielectric charging.

### 3.3. Advancements

Compared with other capacitive RF MEMS switch, as shown in Table 2, the proposed switch in this work shows lower actuation voltage, a significant increase of capacitance ratio and longer lifespan.

## 4. Conclusions

The RF MEMS switch provided in this paper exhibit a high capacitance ratio of 246.3 and a long lifespan of 1.26 × 10^8^ times. On the signal line, a metal–insulator–metal (MIM) capacitor is used to improve the capacitance ratio without taking advantage of a high-dielectric-constant material. The insertion loss is better than 0.5 dB up to 32 GHz, and the isolation is more than 35 dB at the resonant frequency. The achieved lowest actuation voltage of the fabricated switch is 16 V. Because of the outstanding performances, the suggested RF MEMS switch can meet the increased demand for cutting-edge performance in 5G and other high-performance applications.

## Figures and Tables

**Table 1:** The material of each part of the MEMS switch.

Name of Each Part	Material
MEMS beam	Au
MIM floating metallic membrane	Au
Dielectric layer	Si_3_N_4_
CPW transmission line	Au
Substrate	High resistance silicon

**Table 2:** Comparison of developed capacitive RF-MEMS switches.

Author	*C*_up_ (fF)	*C*_down_ (pF)	*C*_r_	Insertion Loss (dB)	Isolation (dB)	Actuation Voltage (V)	Lifespan (Cycles)
Wang L F [13]	-	-	-	0.77@6 GHz	53@6 GHz	15	10^7^
Muhua Li [14]	9.6	0.83	87	0.29@35 GHz	20.5@ 35 GHz	18.3	10^4^
YQ Zhu [15]	-	3.4	-	<1.2@40 GHz	60@35 GHz	0.16	10^5^
Park J [16]	-	-	-	0.29@24 GHz	30.1@24 GHz	25	10^9^
Persano A [17]	-	-	12–16	0.8@25 GHz	20@25 GHz	25–40	10^6^
Yang H H [18]	190	1	5	<1@ DC~20 GHz	11@20 GHz	65	10^4^
MF.B. [19]	-	1.27	-	0.68@40 GHz	35.8@40 GHz	23.6	-
Li-Ya M [20]	140	7.31	52	5.65@40 GHz	24.38@40 GHz	3.04	-
Fouladi [11]	23	2.1	91	0.98@20 GHz	17.9@20 GHz	82	-
This paper	47.1	11.6	246.3	0.5@32 GHz	35@32 GHz	16	10^8^
